# Enhanced In Vitro Recapitulation of In Vivo Liver Regeneration by Co-Culturing Hepatocyte Organoids with Adipose-Derived Mesenchymal Stem Cells, Alleviating Steatosis and Apoptosis in Acute Alcoholic Liver Injury

**DOI:** 10.3390/cells13151303

**Published:** 2024-08-04

**Authors:** Sun A Ock, Seo-Yeon Kim, Young-Im Kim, Won Seok Ju, Poongyeon Lee

**Affiliations:** Animal Biotechnology Division, National Institute of Animal Science, Rural Development Administration, 1500 Kongjwipatjwi-ro, Iseo-myeon, Wanju-gun 55365, Republic of Koreapylee@korea.kr (P.L.)

**Keywords:** hepatocyte organoid, adipose-derived mesenchymal stem cells (A-MSCs), alcoholic liver injury (ALI) model, pig, *CYP1A2*

## Abstract

Hepatocyte organoids (HOs) have superior hepatic functions to cholangiocyte-derived organoids but suffer from shorter lifespans. To counteract this, we co-cultured pig HOs with adipose-derived mesenchymal stem cells (A-MSCs) and performed transcriptome analysis. The results revealed that A-MSCs enhanced the collagen synthesis pathways, which are crucial for maintaining the three-dimensional structure and extracellular matrix synthesis of the organoids. A-MSCs also increased the expression of liver progenitor cell markers (*KRT7*, *SPP1*, *LGR5*^+^, and *TERT*). To explore HOs as a liver disease model, we exposed them to alcohol to create an alcoholic liver injury (ALI) model. The co-culture of HOs with A-MSCs inhibited the apoptosis of hepatocytes and reduced lipid accumulation of HOs. Furthermore, varying ethanol concentrations (0–400 mM) and single-versus-daily exposure to HOs showed that daily exposure significantly increased the level of *PLIN2*, a lipid storage marker, while decreasing *CYP2E1* and increasing *CYP1A2* levels, suggesting that *CYP1A2* may play a critical role in alcohol detoxification during short-term exposure. Moreover, daily alcohol exposure led to excessive lipid accumulation and nuclear fragmentation in HOs cultured alone. These findings indicate that HOs mimic in vivo liver regeneration, establishing them as a valuable model for studying liver diseases, such as ALI.

## 1. Introduction

The liver is essential for detoxification, nutrient synthesis, and blood purification and is the largest vital organ in both humans and animals. Impaired liver function significantly elevates the risk of mortality, often making liver transplantation a necessary treatment. To bridge treatment gaps before transplantation, the use of primary hepatocytes in drug research has been explored, although their short lifespan limits their utility [1]. Recent advances have led to the development of organoids derived from bile duct epithelial cells and hepatocytes. While the former are longer-lasting yet less functional, the latter offer greater efficacy but have limited longevity [2,3,4]. Despite their potential, these solutions face substantial challenges in terms of functionality and durability.

Recent advancements in co-culturing hepatocyte organoids (HOs) with various mesenchymal stem cells (MSCs) have markedly enhanced their structural stability, functionality, size, and lifespan [3,4,5,6]. This innovative technique not only addresses the challenges of in vitro expansion but also shows potential for treating liver failure in both humans and animals, as well as improving in vitro drug testing capabilities. However, the molecular mechanisms underlying these enhancements remain largely unexplored, and further investigation is essential to optimize the therapeutic efficacy of hepatic organoids.

Alcohol consumption is a significant contributor to both acute and chronic liver conditions such as acute alcoholic hepatitis (AAH) and chronic alcoholic liver disease (ALD), leading to severe complications such as liver failure and increased mortality rates [7,8]. Alcohol consumption is responsible for 44% of cirrhosis-related deaths and is the second leading cause of liver transplantation [9]. Therefore, developing precise in vitro models of AAH and chronic ALD is critical for understanding these diseases and exploring new treatment avenues. Moreover, although hepatocyte organoids have effectively modeled non-alcoholic fatty liver disease and have been used in testing therapeutic drugs [10], their use in studying alcohol-induced liver injury remains limited.

This study builds on previous research demonstrating that co-culturing HOs with adipose-derived MSCs (A-MSCs) significantly enhances organoid functionality and longevity. We aimed to elucidate the molecular mechanisms underlying these improvements via transcriptome analysis using the Illumina RNA-Seq platform. Additionally, we evaluated the effects of alcohol exposure, a major risk factor for fatty liver disease and injury, on the functionality of these organoids by examining CYP450 activity, apoptosis, and fat/cholesterol metabolism. This comprehensive approach advances our understanding of the A-MSC-mediated enhancement of HOs, their effects on liver function and lifespan, and the pathological effects of alcohol in liver models.

## 2. Materials and Methods

### 2.1. Ethics Statements

All the experiments were approved by the Institutional Animal Care and Use Committee of the National Institute of Animal Science (approval number: NIAS20212195) of the Rural Development Administration of the Republic of Korea.

### 2.2. Cultivation of HOs

HOs were cultured as previously described by Ock et al. [4]. Briefly, HOs were generated by co-culturing 20,000 primary hepatocytes with 4000 GFP-expressing A-MSCs or alone in 24-well plates with 50 μL Matrigel matrices for 14 days. They were sub-cultured at a 1:2 ratio by enzyme digestion, with fresh A-MSCs added at each passage only for the co-cultured organoids. HOs underwent regular passaging every two weeks throughout the experiment and were maintained in culture for 42 days. Samples were collected at various passages for transcriptomic analysis to evaluate the liver function. The control group included ear fibroblast cells as the negative control, liver tissue from 4.5-month-old male donors as the positive control, and primary hepatocytes as the negative control.

### 2.3. Induction of an ALI Model

The effect of co-culturing A-MSCs with HOs on alcohol-induced liver injury susceptibility was assessed. HOs cultured alone or with MSCs at passage 0 underwent ethanol (Sigma-Aldrich, St. Louis, MO, USA) treatment on day 11 via different protocols: single-dose exposure to 100 mM ethanol for 3 days or daily exposure at doses ranging from 100 mM to 200 mM for 3 days. All experiments were conducted independently. The alcohol content was determined based on previous studies [11,12].

The effects of various alcohol administration methods were also investigated. Briefly, HOs co-cultured with A-MSCs at p0 were subcultured until passage 1 and cultured alone for 11 days. Subsequently, HOs were treated for three more days with either single or daily treatments with ethanol at concentrations of 0, 100, 200, and 400 mM. Each experiment was replicated five times.

### 2.4. Oil Red O Staining

Oil Red O staining revealed neutral triglycerides and lipids in the HOs on day 14 (*p* = 0). Briefly, HOs were fixed with 3.7% formaldehyde (Sigma-Aldrich) for 1 h, rinsed thrice with phosphate-buffered saline (PBS) (Thermo Fisher Scientific, Waltham, MA, USA), and washed once with 60% isopropanol (Sigma-Aldrich). The samples were then stained with 60% Oil Red O solution (Sigma-Aldrich) for 20 min and washed thrice with distilled water. For lipid quantification, Oil Red O dye was eluted from the stained samples with 100% isopropanol, and the absorbance was measured using an ELISA reader. The positive controls utilized a 60% Oil Red O solution, and isopropanol was used as the negative control. This experiment was performed in triplicate. Data are presented as the mean ± standard error of the mean (SEM).

### 2.5. Nile Red Staining

Nile Red staining was performed using a Nile Red Staining Kit (ab228553, Abcam, Cambridge, UK) following the manufacturer’s protocol. Counterstaining was performed using ReadyCount™ Blue Nuclear Stain (Invitrogen™, Thermo Fisher Scientific, Waltham, MA, USA) for 30 min. Samples were then mounted using VECTASHIELD^®^ Antifade Mounting Medium (H-1000; VECTOR, Burlingame, CA, USA) and observed using a confocal microscope (Nikon AX, Tokyo, Japan).

### 2.6. Transcriptome and Differential Gene Expression Analysis

Cells from three separate experiments were merged to yield over 1 µg of RNA for analysis. Transcriptome analysis was performed using an Illumina NovaSeq platform (Illumina, Inc., San Diego, CA, USA) for paired-end sequencing. Adapter sequences and low-quality bases were removed using Trimmomatic v0.38, and alignment to the *Sus scrofa* (Sscrofa11.1) genome was performed using HISAT v2.1.0 [13], incorporating genomic data and gene annotations from the NCBI RefSeq. StringTie v2.1.3b [14,15] was used for transcript assembly and quantification. Differential gene expression was analyzed using edgeR v3.40.2 [16], and TMM normalization was applied to adjust for library size variations. The edgeR exactTest determined significant gene expressions at fold changes ≥ 2 or ≥10 with raw *p*-values < 0.05. PCA confirmed the expression similarities between the samples. Hierarchical clustering and gene enrichment analyses were conducted using gProfiler 2.0 against the GO database, with *p*-values adjusted using the Benjamini–Hochberg method. All data analyses and visualizations were performed using R 4.2.2.

### 2.7. Real-Time Reverse-Transcription Quantitative PCR (Real-Time qPCR)

Total RNA extraction, cDNA synthesis, and real-time qPCR were performed as previously described [4], with modifications. cDNA was synthesized from the purified total RNA (40–500 ng). Specific primer sets were designed to amplify various genes (Table S1). Gene expression was quantified via ΔΔ*CT* analysis, normalized to that of *HPRT1*, and presented as the mean relative quantification (RQ), with error bars reflecting the minimum and maximum RQ values. Each experiment was repeated five times.

### 2.8. Statistical Analyses

Statistical analyses were conducted using Statistical Products and Service Solutions (SPSS) software (ver. 25; International Business Machines Corporation, Armonk, NY, USA), employing Student’s *t*-test for two independent groups and one-way analysis of variance (ANOVA) with post hoc Tukey’s HSD test for three or more groups. Each experiment was replicated five times, with statistical significance set at *p* < 0.05.

## 3. Results

### 3.1. Transcriptome Analysis in HOs

**Figure 1:**
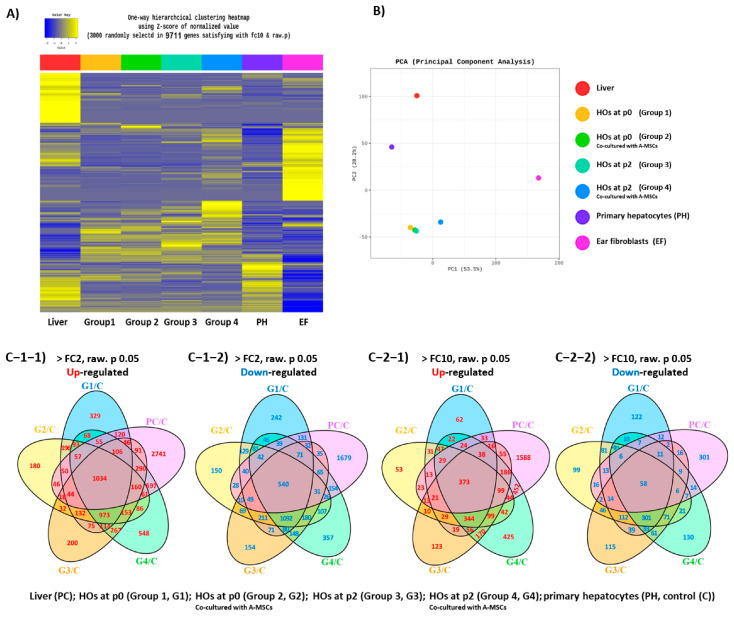
Transcriptomic analysis of porcine primary hepatocyte-derived organoids. (**A**) Heatmap displaying the differentially expressed genes (DEGs) with a 10-fold change cut-off. The groups are divided as follows: at passage 0, 14-day-old hepatocyte organoids (HOs) cultured alone (Group 1, G1) or co-cultured with adipose-derived mesenchymal stem cells (A-MSCs; Group 2, G2), and at passage 2, 42-day-old HOs cultured alone (Group 3, G3) or co-cultured with A-MSCs (Group 4, G4). Liver, primary hepatocytes (PH), and ear fibroblasts (EF) were used as the positive controls (PC), controls (C), and negative controls (NC), respectively; (**B**) Principal Component Analysis (PCA) plot showing inter-group differences; (**C**) Venn diagram analysis of the entire transcriptome, indicating DEGs and showing the number of up- and downregulated genes based on 2-fold (**C-1-1**,**C-1-2**) and 10-fold (**C-2-1**,**C-2-2**) change cut-offs (*p* < 0.05). All groups were analyzed after normalization based on PH, excluding EF.

Initially, RNA sequencing data were analyzed using a 2-fold change cut-off (*p* < 0.05) to identify differentially expressed genes (DEGs). Owing to the high number of DEGs detected, a stricter 10-fold change cut-off (*p* < 0.05) was applied for a more robust identification. The heatmap in Figure 1A displays the DEGs. The liver and ear fibroblast (EF) samples showed similar expression patterns for some transcripts but distinct distributions for most transcripts. Hierarchical clustering analysis indicated that the culture duration and co-culture with A-MSCs notably influenced the gene expression profiles of HOs. Specifically, we observed an increased number of upregulated transcripts in HOs, as indicated by yellow color changes in the heatmap. Principal component analysis (PCA) demonstrated a strong correlation among the HO groups, with 42-day co-cultured HOs in passage 2 (p2) exhibiting a slightly closer correlation with the liver samples than the other groups (Figure 1B). Gene Ontology (GO) analysis revealed significant changes in the biological processes, cellular components, and molecular functions associated with the extracellular matrix and cell adhesion mechanisms, highlighting their crucial role in group differentiation (Figure S1).

Gene expression normalization in primary hepatocytes (PH) was followed by Venn diagram analysis based on the fold-change values calculated at a 2-fold change cut-off and *p* < 0.05 in all groups except EF. Analysis of the up- and downregulation of 20,937 transcripts (Figure 1C) revealed 1034 co-upregulated and 540 co-downregulated transcripts in the liver and HOs groups at a 2-fold change cut-off. Applying the more stringent 10-fold change cut-off identified 373 co-upregulated and 58 co-downregulated transcripts. Venn diagram analysis revealed extensive transcriptome similarities between the liver and 42-day-old co-cultured HOs at p2 (*n* = 452), even at the stricter cut-off. Notably, the liver displayed several unique up- and downregulated transcripts compared to the HO group. Subsequent experiments were performed under these conditions unless otherwise mentioned.

### 3.2. Comprehensive Transcriptomic Analysis of Liver Factors and Metabolism in HOs

**Figure 2:**
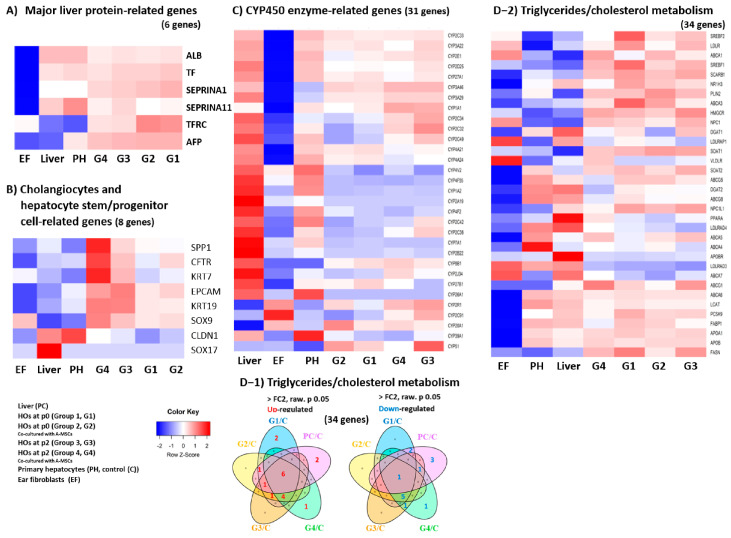
Transcriptomic insights into liver metabolism in HOs. This figure presents heat maps and Venn diagrams to analyze transcripts involved in liver metabolism within HOs. The analyses included transcripts related to hepatic protein synthesis (**A**); cholangiocytes, hepatocyte stem cells, and progenitor cells (**B**); CYP450 enzyme synthesis (**C**); and those involved in triglyceride and cholesterol metabolism, as shown in the Venn diagram (**D-1**) and heatmap (**D-2**). The experimental groups are categorized as follows: G1 (passage 0, 14-day-old HOs cultured alone), G2 (passage 0, 14-day-old HOs co-cultured with A-MSCs), G3 (passage 2, 42-day-old HOs cultured alone), G4 (passage 2, 42-day-old HOs co-cultured with A-MSCs), PH, and EF. A Venn diagram analysis was performed after normalization with PH, excluding EF. All differentially expressed gene analyses were conducted with a 2-fold change cut-off (*p* < 0.05).

Initially, the expression of liver-specific protein synthesis genes, such as alpha-fetoprotein (*AFP*), Serpin Family A Member 1 (*SERPINA1*), and transferrin receptor (*TFRC*), increased in the HOs group but decreased over time (Table 1). Notably, *AFP* expression decreased significantly in the liver group (−6173-fold change [FC]) compared to the HOs group. The albumin synthesis gene *ALB* showed reductions of −4 to −6-FC in the HOs group compared to the liver group. Heatmap analysis of major liver protein transcripts revealed that, except for *AFP*, all other transcripts exhibited expression patterns opposite to those in the EF group, as confirmed in the liver, PH, and HOs groups (Figure 2A).

The expression of liver transcription factors in the HOs group was similar to that in the liver, with slight decreases in Hepatocyte Nuclear Factor 4 Gamma (*HNF4G*) and Krüppel-Like Factor 15 (*KLF15*). In the liver, CCAAT/enhancer-binding protein alpha (*CEBPA*) and Peroxisome Proliferator-Activated Receptor Alpha (*PPARA*) expression increased 2-fold and 5-fold, respectively, compared to those in the HOs group (Table S2).

Levels of apoptosis-related gene transcripts [BCL2 Antagonist/Killer 1 (*BAK1*), BCL2 apoptosis regulator (*BCL2*), BCL2 like 1 (*BCL2L1*), and Cyclin-Dependent Kinase Inhibitor 1A (*CDKN1A*)] increased in the HOs group normalized to PH, in contrast with no change observed in the liver. The mRNA expression of telomerase reverse transcriptase (*TERT*), which is directly linked to cell lifespan, surged over 650-fold in the HOs group, surpassing the 306-fold upregulation in the liver. Remarkably, 42-day-old HOs co-cultured with A-MSCs demonstrated a 5.5-fold increase in TERT levels compared with those of the liver (Table S3).

Eight biliary and hepatic progenitor markers, excluding claudin 1 (*CLDN1*) and SRY-box transcription factor 17 (*SOX17*), were strongly upregulated in 42-day co-cultured HOs compared to both the liver and PH groups (Figure 2B and Table 2). Notably, 42-day-old co-cultured HOs exhibited dramatic increases in keratin 7 (*KRT7*, 1222.52-FC) and Secreted Phosphoprotein 1 (*SPP1*, 254.03-FC) levels. These findings suggest enhanced stem cell potential in co-cultured HOs, as indicated by the upregulation of progenitor markers and increased leucine-rich repeat-containing G protein-coupled receptor 5 (*LGR5*), a marker of gut epithelial stem cells.

Analysis of 31 CYP450 family transcripts, which are key drug metabolism enzymes, revealed that most CYP transcripts were downregulated in HOs compared to those in the PH group (Figure 2C and Table S4). However, *CYP8B1*, *CYP3A29*, and *CYP3A46* were notably upregulated in HOs, particularly in 42-day-old co-cultured HOs.

Venn diagram analysis of 34 genes related to triglycerides and cholesterol metabolism (Figure 2D-1 and Table S5) showed that six transcripts [ATP-binding cassette subfamily A member 7 (*ABCA7*), ATP-binding cassette subfamily G member 1 (*ABCG1*), Fatty Acid Synthase (*FASN*), 3-hydroxy-3-methylglutaryl-CoA Reductase (*HMGCR*), Niemann-Pick C1-Like 1 (*NPC1L1*), and Very Low-Density Lipoprotein Receptor (*VLDLR*)] were upregulated, while ATP-binding cassette subfamily A member 4 (*ABCA4*) was downregulated. Comparing the upregulated genes in the heatmap, *ABCG1* and *NPC1L1* showed similar expression levels in the liver (Figure 2D-2). In the HO group, four transcripts were co-upregulated, five were co-downregulated, two were upregulated, and four were uniquely downregulated in the liver. The 42-day-old co-cultured HOs exhibited the highest similarity to triglycerides and the cholesterol metabolism function of the liver.

In the first stage of the alcohol detoxification process, the key functional genes alcohol dehydrogenase 1C (ADH1C) and alcohol dehydrogenase 4 (ADH4), which convert alcohol to acetaldehyde, were expressed at significantly lower levels than in the liver. In the second stage, the key acetaldehyde dehydrogenase gene, aldehyde dehydrogenase 2 (ALDH2), showed a slight decrease in expression compared to the liver (Table S6).

### 3.3. Transcriptomic Analysis of Key Factors in the 3D Tissue Architecture Formation of HOs

**Figure 3:**
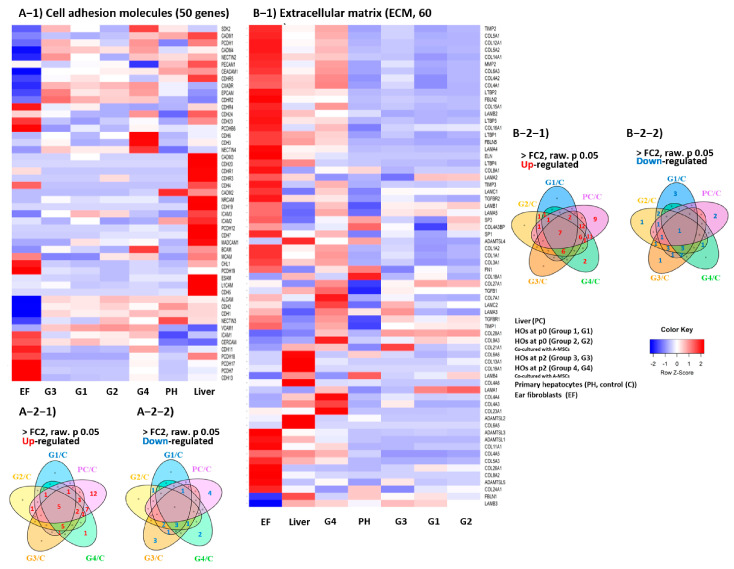
Transcriptomic analysis of the key factors involved in the 3D tissue formation of HOs. This figure presents heat maps and Venn diagrams to analyze the transcripts involved in 3D tissue formation within hepatocyte organoids. (**A-1**) Heatmaps reveal cell adhesion-related transcripts, with Venn diagrams (**A-2-1**,**A-2-2**) showing upregulated and downregulated genes. (**B-1**) Heat maps reveal the expression of genes related to the extracellular matrix, and Venn diagrams (**B-2-1**,**B-2-2**) highlight the upregulated and downregulated genes. The experimental groups are categorized as follows: G1 (passage 0, 14-day-old HOs cultured alone), G2 (passage 0, 14-day-old HOs co-cultured with A-MSCs), G3 (passage 2, 42-day-old HOs cultured alone), G4 (passage 2, 42-day-old HOs co-cultured with A-MSCs), PH, and EF. A Venn diagram analysis was performed after normalization with PH, excluding EF. All differentially expressed gene analyses were conducted with a 2-fold change cut-off (*p* < 0.05).

The heatmap revealed that the mRNA expression of 50 cell–cell adhesion-related genes in 42-day-old co-cultured HOs closely resembled those in the liver (Figure 3A-1). Venn diagram analysis showed that the liver exhibited 33 upregulated (including 12 liver-specific genes), 5 downregulated, and 12 unchanged transcripts compared with PH (Table S7 and Figure 3A-2). Five genes [Cadherin-24 (*CDH24*), Intercellular Adhesion Molecule 1/3 (*ICAM1/3*), Nectin-4 (*NECTIN4*), and Neuronal Cell Adhesion Molecule (*NRCAM*)] were upregulated in all groups. The liver and 42-day-old co-cultured HOs showed the upregulation of 20 shared transcripts (including seven unique to this pair), whereas the liver and 14-day-old HOs showed the fewest shared transcripts (seven transcripts).

Heatmap analysis of 62 extracellular matrix (ECM)-related transcripts revealed that the liver transcriptome closely resembled that of the 42-day co-cultured HOs (Figure 3B-1). Venn diagram analysis revealed the following number of upregulated transcripts: 50 in the liver, 19 in 14-day HOs alone, 24 in 14-day co-cultured HOs, 33 in 14-day HOs alone, and 49 in 42-day co-cultured HOs. The liver and 42-day co-cultured HOs showed 40 common upregulated transcripts (Table S8 and Figure 3B-2). Across all groups, seven transcripts were consistently upregulated [Transforming Growth Factor Beta 1 (*TGF-β1*), tissue inhibitor of metalloproteinases 1 (*TIMP1*), ADAMTS-like 5 (*ADAMTSL5*), Collagen Type 16A1 (*COL16A1*), *COL21A1*, *COL27A1*, and Calmodulin-2 (*CAMC2*)], while only one transcript, *COL18A1*, was consistently downregulated.

Transcripts crucial for 3D tissue architecture, notably those regulating cell–cell adhesion and the ECM, were markedly upregulated in 42-day-old co-cultured HOs, confirming the pivotal role of A-MSCs in hepatocyte organoid development.

### 3.4. Effect of A-MSCs Co-Culture on Ethanol-Induced Hepatic Lipid Accumulation in HOs

HOs alone and HOs co-cultured with A-MSCs were treated with a single dose of 100 mM ethanol for 3 days. The expression of lipogenesis and cholesterol esterification markers, including apolipoprotein B (*APOB*), low-density lipoprotein receptor (*LDLR*)1, sterol regulatory element binding transcription factor (*SREBF*) 1, perilipin 2 (*PLIN2*), fatty acid binding protein 1 (*FABP1*), and fatty acid synthase (*FASN*), was analyzed (Figure S2). We found that *APOB*, *LDLR1*, *SREBF1*, and *PLIN* were upregulated in the alcohol-treated HO groups, while PLIN expression notably increased 1.9-fold in the HO-alone group. Therefore, these genes were selected as indicators of lipogenesis and cholesterol esterification for future experiments.

**Figure 4:**
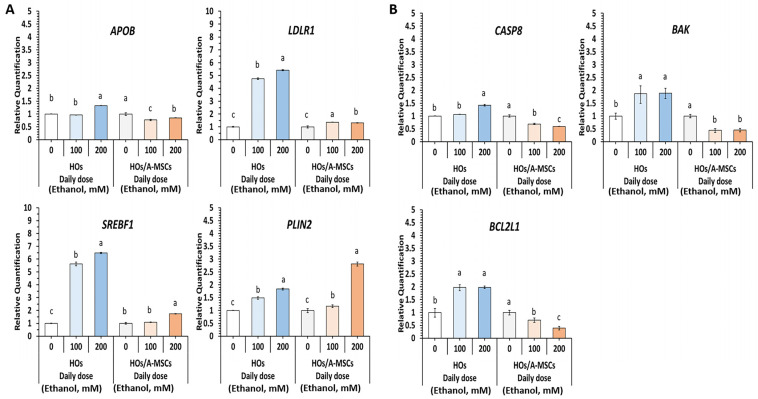
Effects of ethanol treatment on gene expression in co-cultures of HOs and A-MSCs. On day 11, HOs were cultured with a single or daily dose of 0, 100, or 200 mM ethanol for three days. (**A**) Genes related to lipid metabolism included *APOB*, *LDLR1*, *SREBF1*, and *PLIN2*; (**B**) Genes related to apoptotic pathways included *CASP8*, *BAK*, and *BCL2L1*. The data are presented as the mean relative quantification (RQ) ± the maximum and minimum values, normalized to HOs without ethanol for RQ in each of the single and daily treatment groups. Statistical significance was assessed using a one-way analysis of variance (ANOVA) with ^a–c^ *p* < 0.05 set as the threshold after five repetitions.

The effects of daily exposure to varying ethanol concentrations on HOs were investigated over a three-day period. In HOs alone, *LDLR1* and *SREBF1* expression levels increased with the alcohol dose. Co-cultured HOs showed reduced sensitivity to *APOB*, *LDLR1*, and *SREBF1* compared with HOs alone, with peak expression at 200 mM alcohol for most genes. Interestingly, the apoptosis-related genes *CASP8*, *BAK*, and *BCL2L1* were notably downregulated in co-cultured HOs, unlike in HOs alone. Two-dimensionally cultured A-MSCs without HOs showed modestly reduced APOB, SREBF1, and LDLR1 levels (*p* < 0.05), and no effect on *PLIN2* was observed (*p* > 0.05) under daily 200 mM ethanol exposure (Figure 4).

**Figure 5:**
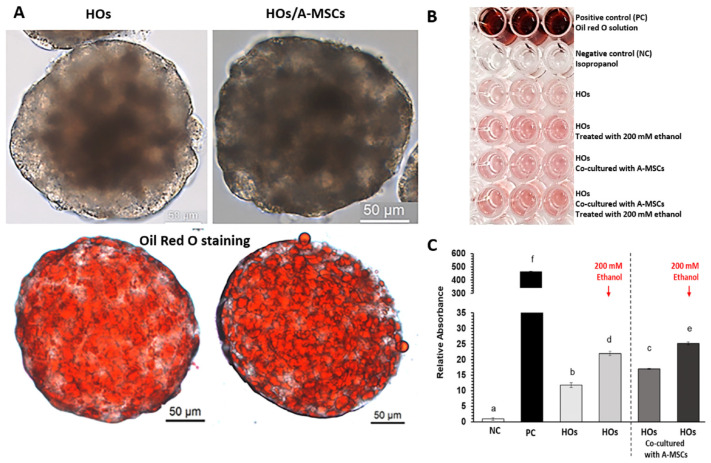
Absorbance analysis after Oil Red O staining in HOs co-cultured with A-MSCs under ethanol exposure. On day 11, HOs co-cultured with A-MSCs or alone were exposed to 200 mM ethanol for three days. (**A**) Representative images of HOs before and after Oil Red O staining (ethanol-unexposed control). (**B**) Samples were prepared for absorbance measurements after eluting Oil Red O from each sample. The PC was 60% Oil Red O, while the NC was 100% isopropanol. (**C**) The quantified absorbance values were normalized to the NC. Data represent the mean ± SEM (n = 3). Statistical significance was determined using one-way ANOVA (^a–f^ *p* < 0.05; three replicates).

Lipid accumulation in HOs was quantified by measuring the absorbance of Oil Red O dye. Before ethanol treatment, co-cultured HOs had a 1.4-fold higher absorbance than HOs alone. After treatment with 200 mM ethanol, HOs alone showed a 1.9-fold increase, whereas the co-cultured HOs showed a 1.5-fold increase (Figure 5). A-MSCs may not directly metabolize alcohol-induced lipids and cholesterol; however, co-culturing them with HOs reduces lipid and cholesterol build-up, suggesting a supportive role for managing lipid and cholesterol metabolism in HOs.

### 3.5. Evaluation of Alcohol-Induced Liver Injury Models Using Diverse Ethanol Exposure Methods in HOs

**Figure 6:**
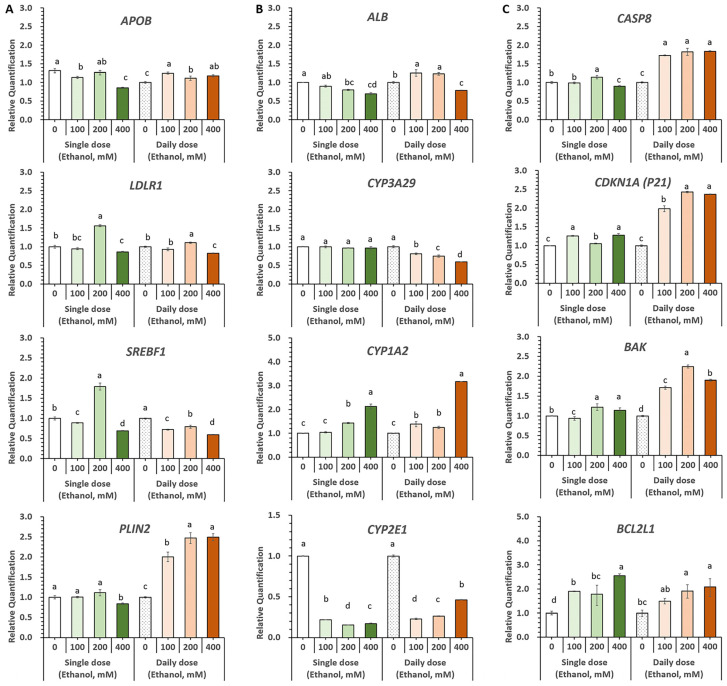
Evaluation of the alcohol-induced hepatocyte organoid injury model under various alcohol treatments. HOs were co-cultured with A-MSCs for 14 days (passage 0) and subcultured for 11 days without A-MSCs before exposure to various alcohol concentrations for 3 days. HOs received either a single dose (on day 1) or daily replacements of ethanol-supplemented medium (0, 100, 200, 400 mM) for 3 days. The genes investigated include (**A**) those involved in lipid metabolism (*APOB*, *LDLR1*, *SREBF1*, *PLIN2*), (**B**) those associated with albumin and cytochrome P450 (CYP) enzymes (*ALB*, *CYP3A29*, *CYP1A2*, *CYP2E1*), and (**C**) those involved in the apoptosis pathway (*CASP8*, *CDKN1A*, *BAK*, *BCL2L1*). Data are presented as the mean relative quantification ± the maximum and minimum values and normalized to that of the ethanol-untreated HOs for each treatment group. Statistical significance was assessed using one-way analysis of variance (ANOVA) with ^a–d^ *p* < 0.05 as the threshold after five repetitions.

Healthy HOs were evaluated as a model for alcohol-induced liver injury by testing different ethanol concentrations (0–400 mM) and administration methods (single vs. daily), followed by analyzing the expression of genes associated with lipid/cholesterol metabolism, albumin synthesis, detoxification, and cell death (Figure 6).

Genes related to lipid and cholesterol metabolism were influenced more by the administration method than by ethanol concentration (Figure 6A). A single dose of 400 mM ethanol decreased the expression of all four genes, whereas a lower dose of 200 mM ethanol increased *LDLR1* expression by 1.6-fold and *SREBF1* expression by 1.8-fold. Daily treatment with 400 mM ethanol decreased *LDLR1* and *SREBF1* levels, while 200 mM ethanol increased *APOB*, *LDLR1*, and *PLIN2* levels.

*ALB* levels decreased with both single and daily 400 mM ethanol treatments. *CYP3A29* expression did not change with a single dose but decreased in a concentration-dependent manner with daily ethanol treatment. *CYP1A2* expression increased with both single and daily treatments, peaking at 400 mM ethanol. The expression of *CYP2E1*, which is involved in alcohol metabolism, was significantly decreased by both the single and daily ethanol treatments. However, there was a slight concentration-dependent increase in *CYP2E1* expression in the daily treatment group despite lower expression than in the 0 mM group (Figure 6B).

**Figure 7:**
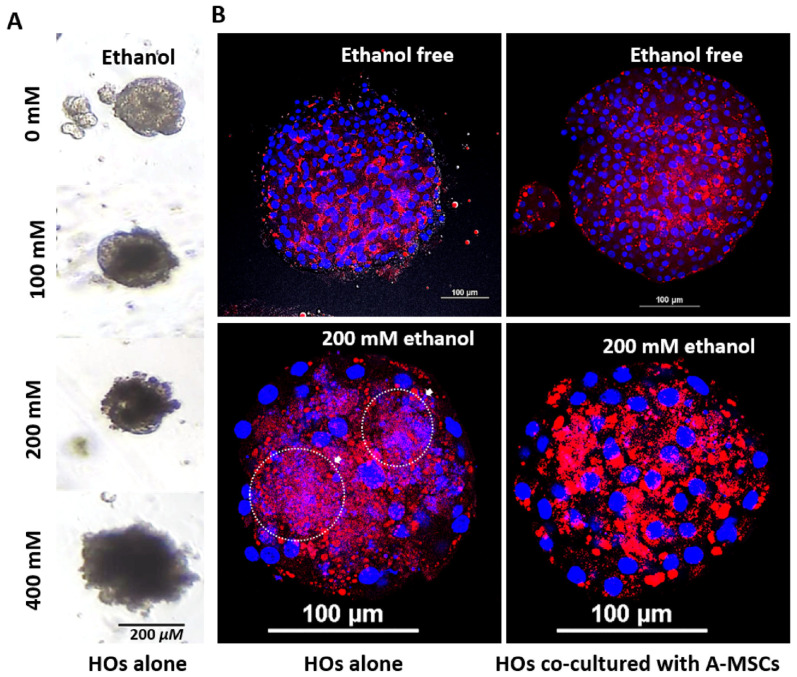
Assessing alcohol-induced hepatic steatosis in HOs. HOs were co-cultured with A-MSCs for 14 days (passage 0) and subcultured for 11 days without A-MSCs before exposure to various alcohol concentrations (0–400 mM) for 3 days. (**A**) Morphological changes in HOs exposed to various ethanol concentrations. Scale bars represent 200 μm. On day 25, HOs alone or co-cultured with A-MSCs were incubated in fresh culture medium supplemented with 200 mM ethanol for 3 days. The sections were stained with a Nile Red solution, counterstained with DAPI, and observed under a confocal microscope. (**B**) The top images show untreated HOs (left: HOs cultured alone, right: HOs co-cultured with A-MSCs), whereas the bottom images show HOs treated with 200 mM ethanol (left: HOs cultured alone, right: HOs co-cultured with A-MSCs). Red, neutral lipids; blue, nuclei; white arrows, fragmented nuclei. Scale bars represent 100 μm. White dashed circles indicate areas where the accumulated fat was distributed along the fragmented nuclei. White dashed circles highlighted by arrows indicate areas where accumulated fat was found alongside fragmented nuclei.

Compared to the single-dose treatment, daily ethanol treatment resulted in faster and more pronounced upregulation of the apoptosis-related genes *CASP8*, *CDKN1A*, *BAK*, and *BCL2L1*, starting at 100 mM (Figure 6C). Moreover, daily exposure to ethanol significantly changed the shape of HOs, causing some cells to die when the concentration reached 100 mM and most cells to die at 400 mM (Figure 7A).

Nile Red staining revealed that HOs exhibited a stronger structure with evenly distributed lipids and intact edges when co-cultured with A-MSCs than when cultured alone. When cultured alone and exposed to 200 mM ethanol, HOs accumulated a large amount of lipids, and their nuclei were fragmented in the lipid-rich areas; however, co-culture with A-MSCs allowed their nuclei to remain intact while having relatively lower lipid accumulation (Figure 7B).

In summary, daily exposure to ethanol concentrations exceeding 100 mM rapidly activates lipid metabolism and induces cell death, leading to a notable increase in *CYP1A2* levels in HOs.

## 4. Discussion

This study explored how co-culture with A-MSCs extends HOs’ longevity and enhances their hepatocyte progenitor capacity. Transcriptomic analysis showed that 42-day-old HOs co-cultured with A-MSCs exhibited gene expression profiles that closely resembled those of native livers. GO and DEG analyses further highlighted the involvement of these organoids in ECM remodeling and cell adhesion, which are essential for maintaining the structural integrity of the liver. To assess the feasibility of using HOs as a model for alcohol-induced liver injury, we investigated the protective effects of an A-MSC co-culture against alcohol exposure. By employing varying alcohol concentrations, we confirmed that HOs could effectively simulate acute alcohol-induced liver injury.

Our transcriptome analysis confirmed that HOs effectively replicated the liver physiology, synthesizing key proteins that function as essential drug-metabolizing enzymes. Despite the slight downregulation of *ALB* in HOs compared to PH, which was likely due to the in vitro conditions, the significant > 8-fold increase in the expression of *AFP*, a marker for fetal liver, liver regeneration, and cancer [3,17] in 14-day-old HOs, suggests a regenerative process akin to in vivo liver regeneration. This elevation may also be influenced by the fact that the PH were derived from approximately one-month-old pigs. Additionally, our findings confirmed our hypothesis by demonstrating an increase in the expression of *TERT*, a key factor in extended cellular lifespan, in HOs compared to the liver. Despite the overall decrease in cytochrome P450 (CYP) enzyme synthesis, the expression of key porcine xenobiotic metabolic genes such as *CYP3A29* and *CYP3A46*, which are equivalent to that of human *CYP3A4* [18,19], increased significantly during culture. This consistent trend, as previously noted [4], underscores the suitability of HOs as models to investigate xenobiotic metabolism. After 42 days of culture, regardless of co-culture with A-MSCs, *CYP3A46* levels in HOs approached those in the liver. This confirms the value of our HOs for drug hepatotoxicity analysis and highlights the necessity for further exploration of the function of *CYP3A46*, which is relatively less understood than that of *CYP3A29*.

HOs can enhance the limited functionality of cholangiocyte-derived organoids; however, their bile secretion remains constrained. Although certain hepatocytes can differentiate into both hepatocytes and duct-forming cells [20], fetal HOs have demonstrated partial functionality by expressing Multidrug Resistance-Associated Protein 2 (MRP2), which facilitates the transport of substances such as drugs and toxins from the hepatocytes to the bile [3]. Our investigation revealed an increased expression of transcripts associated with potential bipotent biliary and progenitor markers in HOs. Specifically, six of the eight factors critical for biliary function and liver stem/progenitor cells were upregulated in 42-day-old HOs. Co-culture with A-MSCs significantly increased their expression, such as with *KRT7*, which is associated with liver regeneration, and with *SPP1*, which regulates *MRP2* expression and osteopontin synthesis, and is involved in liver regeneration upon injury. This confirms that our HOs possess the maturation, differentiation, and regenerative potential inherent to the native liver.

Creating structurally stable mini-organs, such as HOs, from a single cell requires a crucial interplay between the extracellular matrix (ECM), which provides structure, scaffolding, and cell polarization [21], and cadherin/cell adhesion molecules, which facilitate cell adhesion, intercellular communication, and movement. The ECM of the liver, which comprises 10% of its composition [22], is primarily composed of collagen, which accounts for 30% of the ECM of the liver [21]. Our GO and DEG analyses revealed enhanced collagen synthesis in HOs. Despite the observed increase in the expression of Matrix Metallopeptidase 2 (*MMP2*), which is responsible for collagen degradation, the significant effect of co-culture conditions was evident as they effectively mimicked in vivo liver regeneration in HOs by upregulating TGFβ expression, promoting ECM accumulation, and elevating TIMP levels, which inhibit ECM degradation.

The protective effects of A-MSCs co-cultured with HOs against liver lipid accumulation and damage were assessed under alcohol-induced liver injury. This co-culture significantly improved liver function and reduced lipid accumulation and cell death in HOs. The exposure of HOs to 200 mM alcohol for three days, along with A-MSC co-culture, alleviated fat accumulation and cell death. This observation is consistent with previously reported evidence that MSC-derived extracellular vesicles can suppress fatty acid uptake and enhance lipid metabolism by upregulating fatty acid beta-oxidation genes, exerting an anti-steatogenic effect [23]. Additionally, MSCs alleviate oxidative stress and promote tissue regeneration by secreting Tumor Necrosis Factor-Stimulated Gene 6 and other growth factors [23,24,25].

To accurately model alcohol-induced liver injury, we initially cultured healthy HOs with robust fat and liver metabolic functions in a co-culture setup. Subsequently, we transitioned them to a single culture to directly assess the progression of alcohol-induced liver injury under various alcohol exposure conditions. Contrary to expectations, a single exposure to 100mM ethanol did not significantly impact lipid cholesterol metabolism and cell death. This may be due to species-specific differences, higher fat content in pig organoids, or variations in organoid generation methods like the use of Matrigel. Daily ethanol dosing upregulated the expression of crucial genes, such as *APOB* and *PLIN2*, enhancing liver fat transport and metabolism. Additionally, increased ethanol concentration correlated with an increased expression of cell death-related genes, starting at 100 mM, along with decreased levels of the detoxification enzyme *CYP3A24*, indicating cell damage. Surprisingly, the expression of CYP2E1, a key enzyme in alcohol-induced oxidative stress and steatosis, decreased, in contrast to previous reports suggesting its role primarily in chronic alcohol consumption [26,27]. Conversely, *CYP1A2* expression showed an alcohol concentration-dependent increase, consistent with previous findings suggesting that abnormal lipid metabolism induces hepatic steatosis [11,28]. These observations confirm that our HOs mimic an acute alcohol-induced liver injury model, as confirmed by Nile Red staining, revealing nuclear fragmentation due to apoptosis in areas of excessive lipid accumulation.

## 5. Conclusions

Our study revealed that co-culturing HOs with A-MSCs substantially enhances their applicability in liver disease research, including the simulation of acute alcohol-induced liver injury. A-MSCs facilitate ECM development by synthesizing collagen, stabilizing the liver structure, prolonging the organoid lifespan, and promoting the differentiation of hepatocytes into bipotent cells. Our findings underscore the potential of A-MSCs to support the regeneration of diverse liver cell types and offer promising implications for liver disease treatment and research. Furthermore, HOs co-cultured with A-MSCs exhibited increased *KRT7*/*SPP1* expression, indicating their potential as in vivo liver regeneration models. Our findings also suggest that CYP1A2 plays a more critical role than CYP2E1 in acute alcohol-induced fatty liver disease. Overall, our results highlight the value of HOs as a tool for drug screening and the development of novel therapies and suggest that A-MSCs may contribute to inhibiting cell death and alleviating lipid accumulation in alcoholic fatty liver injury. Additionally, the limited 14-day monitoring of Matrigel-based HOs is unsuitable for chronic liver disease modeling due to the limitations of long-term monitoring and HO size. Therefore, a new experimental model for long-term liver drug toxicity evaluation using HOs must be urgently developed.

## Figures and Tables

**Table 1:** Expression of key transcripts in liver protein synthesis in HOs.

Genes		* Fold-Change Values [log_2_ Each Group/Primary Hepatocytes, (PH)]				
		Group 1	Group 2	Group 3	Group 4	Liver
1	AFP	**8.71**	**8.82**	**6.74**	**4.65**	**−6173.24**
2	ALB	**−4.01**	**−4.27**	**−3.89**	**−5.98**	1.07
3	SERPINA1	**10.77**	**7.20**	**5.20**	**3.55**	1.02
4	SERPINA11	**−5.96**	**−8.12**	**−3.59**	**−5.63**	**−2.85**
5	TF	1.69	1.20	1.19	−1.45	−1.43
6	TFRC	**5.31**	**5.64**	**3.46**	**3.36**	1.19

* For genes exhibiting a fold change greater than 2, the *p*-value was less than 0.05. Red and blue indicate the upregulation and downregulation of genes, respectively.

**Table 2:** Expression analysis of biliary epithelial, hepatocyte progenitor, and digestive tract stem cell factors in HOs.

Genes		* Fold-Change Values [log_2_ Each Group/Primary Hepatocytes, (PH)]				
		Group 1	Group 2	Group 3	Group 4	Liver
1	CFTR	1.38	**3.30**	**4.27**	**19.89**	**−1.13**
2	CLDN1	**−6.74**	**−4.77**	**−3.81**	**−3.18**	**−1.49**
3	EPCAM	**2.02**	**2.77**	**9.60**	**5.84**	**−5.47**
4	KRT19	**2.28**	**2.27**	**9.15**	**9.72**	**−2.83**
5	KRT7	**28.32**	**47.55**	**190.98**	**1222.52**	**15.77**
6	SOX9	**6.63**	**7.46**	**9.67**	**18.48**	**−2.05**
7	SOX17	1.00	1.00	1.00	1.00	**350.65**
8	SPP1	**15.74**	**12.83**	**27.11**	**254.03**	**10.20**
9	LGR5	−1.38	**−2.27**	1.19	**4.36**	**−2.02**

* For genes exhibiting a fold change greater than 2, the *p*-value was less than 0.05. Red and blue indicate the upregulation and downregulation of genes, respectively.

## Data Availability

All data generated or analyzed during this study are included in this published article and its Supplementary Materials.

## Supplementary Materials

The following supporting information can be downloaded at https://www.mdpi.com/article/10.3390/cells13151303/s1. Table S1: primer sequences for target gene amplification; Table S2: expression profiles of liver transcription factors in hepatocyte organoids (HOs); Table S3: transcript expression patterns during the lifespan and proliferation of hepatocyte organoids (HOs); Table S4: metabolic transcript expression patterns of CYP enzymes in hepatocyte organoids (HOs); Table S5: transcript expression patterns of triglyceride and cholesterol metabolism in hepatocyte organoids (HOs); Table S6: expression profiles of alcohol-degrading transcripts in hepatocyte organoids (HOs); Table S7: transcript expression patterns of cadherins and cell adhesion molecules in hepatocyte organoids (HOs); Table S8: extracellular matrix transcript expression patterns in hepatocyte organoids (HOs); Figure S1: Gene Ontology (GO) analysis results of hepatocyte organoids (HOs); Figure S2: pathway analysis of lipid and cell death responses to single-dose ethanol exposure in hepatocyte organoids (HOs); Figure S3: effect of ethanol exposure on the expression of lipid metabolism and apoptosis genes in A-MSCs.
