# Supplementary material for: Enhanced In Vitro Recapitulation of In Vivo Liver Regeneration by Co-Culturing Hepatocyte Organoids with Adipose-Derived Mesenchymal Stem Cells, Alleviating Steatosis and Apoptosis in Acute Alcoholic Liver Injury
Source: Cells. 2024 Aug 4;13(15):1303. doi: 10.3390/cells13151303 (PMC11311897; doi:10.3390/cells13151303)
Supplement: Supplementary file 1 [file cells-13-01303-s001.zip › cells-3100490-supplementary.pdf]

Table S1. Primer sequences for target gene amplification.

Gene	Primer sequences	GenBank accession number
Apolipoprotein B (APOB)	F-CACCAAAACGGAAGCGATTC<br>R-CAGCGGGTAGTAGGAGGAAGAG	NM_001375388.1
Low density lipoprotein receptor (LDLR)	F-CCCAGCTTTTCCTCCTATGACA<br>R-CCAGTATATGTTGCTGTGGATCCA	NM_001206354.2
Sterol regulatory element binding transcription factor 1 (SREBF1)	F -CGGACGGCTCACAATGC<br>R -TCGATGATCTTGTCATTGATGGA	NM_214157.1
Perilipin 2 (PLIN2)	F-ACTGGCTGGTAGGTCCCTTTTAT<br>R-CCCGGGACTGAGCATCCT	NM_214200
Fatty acid binding protein 1(FABP1)	F -TCCAAGGTCGTCCAGAATGAG<br>R -CCCAGTCAGGGTCTCCATCTC	NM_001004046.2
Fatty acid synthase (FASN)	F-TCCAAGGTCGTCCAGAATGAG<br>R-CCCAGTCAGGGTCTCCATCTC	NM_001099930.1
Albumin (ALB)	F-GAAGACACCAGTGAGCGAAAAA<br>R-AAGCAAGGCCGTCTGTTCA	NM_001005208.1
Cytochrome P450 family 3 subfamily A member 29 (CYP3A29)	F-AGGAGGAGATTGAGGCAACTTTC<br>R-TCTGTGCCAGGGCATCGTA	NM_214423.1
Cytochrome P450 family 1 subfamily A member 2 (CYP1A2)	F-GGCAGGGCGACGATTTC<br>R-TCAGTGACCAGAGTGAAGCTGTAGA	NM_001159614.1
Cytochrome P450 family 2 subfamily E member 1(CYP2E1)	F-GCAGGAAAGCGGGTGTGT<br>R-CAGCCATGAACASGAACAGTTC	NM_214421.1
CDKN1A cyclin dependent kinase inhibitor 1A (CDKN1A, p21)	F-CCTCCCAGGGCAGGAAA<br>R-GCGTTTGGAGTGGTAGAAATCTG	XM_013977858.2
Caspase 8 (CASP8)	F-GGGAAGCCCCCATCTATGA<br>R-CCCTTGACAAGCCTGAATGAA	NM_001031779
BCL2 antagonist/killer 1 (BAK1)	F-TCACCCTGCCCCTAGAACCT<br>R-CTGGAATTCCGAGTCGTATCG	XM_001928147.2
BCL2 apoptosis regulator (BCL2l1)	F-GGTACCGG GGGCATTCAGT<br>R-TCCCGGAAGAGTTCGTTCAA	NM_214285.1
Hypoxanthine phosphoribosyltransferase 1 (HPRT1)	F-CGGCTTGCTCGAGATGTGAT<br>R-AGCACACAGAGGGCTACGATGT	NM_001032376.2

Table S2. Expression profiles of liver transcription factors in hepatocyte organoids

Genes		Fold change values [log_2 Each group/primary hepatocytes, (PH)]				
		Group 1	Group 2	Group 3	Group 4	Liver
1	CEBPA	1.55	1.13	1.10	-1.67	**2.31**
2	FOXA1	-1.13	-1.18	1.21	-1.46	1.38
3	FOXA2	-1.16	-1.87	**-2.22**	**-2.40**	-1.76
4	FOXA3	**2.42**	1.86	1.90	1.28	1.26
5	GATA6	1.39	1.55	1.81	1.69	1.72
6	HNF1A	1.38	-1.07	-1.32	-1.65	1.80
7	HNF1B	1.09	-1.67	-1.02	-1.03	**-6.65**
8	HNF4A	1.78	1.35	1.39	1.20	1.84
9	HNF4G	**-6.15**	**-3.92**	**-5.77**	**-7.32**	**-2.07**
10	KLF15	**-2.23**	**-2.44**	**-3.16**	**-3.89**	1.85
11	PPARA	1.07	-1.10	1.20	1.56	**4.69**

*For genes exhibiting a fold change greater than 2, the p-value was less than 0.05.

Table S3. Transcript expression patterns during the lifespan and proliferation of hepatocyte organoids

Genes		Fold change values [$\log_2$ Each group/primary hepatocytes, (PH)]				
		Group 1	Group 2	Group 3	Group 4	Liver
1	BAK1	**4.52**	**4.48**	**5.78**	**6.01**	1.20
2	BCL2	**3.80**	**3.95**	**6.05**	**4.22**	1.00
3	BCL2L1	**4.63**	**4.47**	**6.39**	**8.65**	-1.26
4	CDKN1A	**3.25**	**2.72**	**3.95**	**4.67**	1.26
5	TEP1	**2.81**	**3.42**	**3.01**	**4.35**	1.21
6	TERT	**650.24**	**776.32**	**1479.02**	**1693.45**	**306.01**
7	TP53	**3.69**	**3.06**	1.93	**2.08**	1.19

* For genes exhibiting a fold change greater than 2, the p-value was less than 0.05.

Table S4. Metabolic transcript expression patterns of CYP enzymes in hepatocyte organoids

Genes		Fold change values [$\log_2$ Each group/primary hepatocytes, (PH)]				
		Group 1	Group 2	Group 3	Group 4	Liver
1	CYP1A1	-1.19	-1.68	**2.54**	**3.07**	**-1.43**
2	CYP1A2	**-279.86**	**-316.98**	**-166.52**	**-140.98**	**8.38**
3	CYP20A1	1.62	**2.67**	1.57	1.75	**-2.88**
4	CYP26A1	**-1856.92**	**-2290.80**	**-2261.41**	**-2266.31**	**-1.17**
5	CYP27A1	-1.72	**-2.50**	-2.00	**-3.96**	**2.3**
6	CYP27B1	1.10	-1.01	-1.27	-1.05	**1.54**
7	CYP2A19	**-1101.12**	**-33.88**	**-1340.88**	**-1343.78**	**2155.73**
8	CYP2B22	**-4.78**	**-5.00**	**-5.18**	**-2.34**	**81.73**
9	CYP2C32	**-3.19**	**-9.80**	**4.87**	1.56	**7.03**
10	CYP2C33	**-3.21**	**-2.47**	**-2.45**	**-2.68**	**1.07**
11	CYP2C34	1.07	-1.77	**6.50**	**2.94**	**19.21**
12	CYP2C36	**-4.96**	**-7.25**	1.31	-1.98	**9.38**
13	CYP2C42	**-9.87**	**-22.56**	**-2.80**	**-8.26**	**1.49**
14	CYP2C49	**-11.46**	**-28.92**	1.00	**-2.86**	**9.54**
15	CYP2C91	1.03	**-2.68**	**4.52**	**2.44**	**-1.38**
16	CYP2D25	**-3.16**	**-3.81**	-1.88	**-3.48**	**3.79**
17	CYP2E1	**-3.97**	**-9.59**	**-2.20**	**-2.70**	**1.55**
18	CYP2J34	-1.54	**-2.02**	1.19	**2.04**	**9.90**
19	CYP2R1	1.49	2.00	**2.60**	**2.27**	**-1.56**
20	CYP39A1	**-3.98**	**-3.14**	**-3.09**	**-4.48**	**-1.90**
21	CYP3A22	-1.71	**-2.11**	-1.12	**-3.95**	**2.73**
22	CYP3A29	**4.37**	**4.33**	**6.05**	**6.55**	**25.90**
23	CYP3A46	**11.20**	**12.79**	**22.47**	**23.07**	**20.93**
24	CYP4A21	-1.27	-1.49	-1.92	**-2.81**	**2.46**
25	CYP4A24	**-2.11**	**-2.54**	**-3.18**	**-4.69**	**1.66**
26	CYP4F2	**-13.48**	**-6.62**	**-10.54**	**-24.55**	**4.38**
27	CYP4F55	**-22.32**	**-18.43**	**-18.19**	**-23.30**	**2.96**
28	CYP4V2	**-33.66**	**-13.68**	**-28.89**	**-10.93**	**1.22**
29	CYP51	**2.23**	**2.68**	**3.31**	1.90	**1.44**
30	CYP7A1	**-27.47**	**-2363.33**	**-112.67**	**-2338.06**	**19.20**
31	CYP8B1	**955.77**	**893.59**	**1032.33**	**1312.98**	**2880.03**

* For genes exhibiting a fold change greater than 2, the p-value was less than 0.05.

Table S5. Transcript expression patterns of triglyceride and cholesterol metabolism in hepatocyte organoids

Genes		Fold change values [$\log_2$ Each group/primary hepatocytes, (PH)]				
		Group 1	Group 2	Group 3	Group 4	Liver
1	ABCA1	1.36	1.98	1.57	**2.15**	-1.71
2	ABCA3	**2.50**	1.99	1.49	1.55	1.15
3	ABCA4	**-7.43**	**-5.59**	**-10.38**	**-6.51**	**-8.90**
4	ABCA5	**-2.45**	-1.66	-1.37	-1.34	**-2.37**
5	ABCA6	-1.75	-1.87	-1.88	**-2.13**	-1.27
6	ABCA7	**169.04**	**59.64**	**329.45**	**525.79**	**1816.20**
7	ABCG1	**117.23**	**71.73**	**207.01**	**270.06**	**77.68**
8	ABCG5	-1.77	-1.73	**-2.69**	**-7.11**	**-2.56**
9	ABCG8	**-3.22**	**-5.52**	**-4.42**	**-7.31**	-1.35
10	APOA1	1.59	-1.28	1.19	-1.09	**-2.14**
11	APOB	**-2.00**	-1.45	-1.50	-1.30	**-2.61**
12	APOBR	**-26.19**(p>0.05)	-1.56	1.48	-1.54	**79.80**
13	DGAT1	-1.03	-1.97	1.14	-1.29	1.86
14	DGAT2	**-4.28**	**-5.50**	**-6.73**	**-20.29**	1.44
15	FASN	**15.96**	**6.10**	**12.79**	**9.65**	**2.30**
16	FABP1	-1.20	-1.75	**-4.10**	**-6.93**	-1.21
17	HMGCR	**25.55**	**19.50**	**42.99**	**30.16**	**2.02**
18	LCAT	**-2.72**	**-3.51**	**-3.34**	**-3.86**	-1.66
19	LDLR	**3.59**	**2.46**	**3.14**	**2.57**	1.97
20	LDLRAD3	**-9.61**	**-9.69**	**-16.43**	**-6.42**	1.21
21	LDLRAD4	**-3.52**	**-4.28**	**-6.61**	**-2.71**	1.49
22	LDLRAP1	**2.45**	**2.13**	1.63	1.87	**3.67**
23	NPC1	**5.56**	**4.53**	**7.25**	**6.26**	-1.08
24	NPC1L1	**33.47**	**22.02**	**31.35**	**9.93**	**15.30**
25	NR1H3	1.59	1.38	1.25	-1.01	1.39
26	PCSK9	**4.36**	**2.07**	**2.20**	1.25	**2.66**
27	PLIN2	1.37	1.78	1.39	1.34	**-3.05**
28	PPARA	1.07	-1.10	1.20	1.56	**4.69**
29	SCARB1	**2.09**	1.71	1.97	1.72	-1.32
30	SOAT1	**2.51**	**3.00**	**2.85**	**2.32**	**-2.67**
31	SOAT2	1.29	-1.21	1.59	1.00	**-4.10**
32	SREBF1	**7.31**	**3.45**	**4.43**	**4.30**	-1.36
33	SREBF2	**3.45**	**2.21**	**2.46**	1.99	1.50
34	VLDLR	**37.36**	**39.43**	**61.19**	**194.86**	**65.72**

*Genes with fold changes greater than 2 were labeled with "p > 0.05" next to the gene name if their p-value exceeded 0.05

Table S6. Expression profiles of alcohol-degrading transcripts in hepatocyte organoids

Genes		Fold change values [$\log_2$ Each group/primary hepatocytes, (PH)]				
		Group 1	Group 2	Group 3	Group 4	Liver
1	ADH1C	**-17.43**	**-15.21**	**-78.33**	**-33.99**	-1.15
2	ADH4	**-20.77**	**-12.89**	**-12.82**	**-18.88**	**4.14**
3	ADH5	**-2.01**	**-2.21**	**-2.02**	**-2.68**	**-3.39**
4	ADHFE1	**-2.98**	**-2.95**	**-6.40**	**-6.97**	**2.41**
5	ALDH1A1	**2.13**	**2.48**	**3.12**	**2.77**	1.38
6	ALDH1A2	**-2.31**	-1.92	-1.88	-1.26	**8.65**
7	ALDH1A3	-1.89	**-73.53**	1.10	**3.08**	**6.30**
8	ALDH1B1	-1.35	-1.49	-1.20	-1.94	**-2.92**
9	ALDH1L1	1.91	1.31	**2.15**	-1.02	**5.92**
10	ALDH1L2	**3.93**	**4.67**	**2.21**	**2.28**	-3.66
11	ALDH2	-1.80	**-2.08**	**-2.11**	**-2.92**	1.29
12	ALDH3A1	-1.16	-1.27	-1.94	1.21	**-174.04**
13	ALDH3A2	-1.59	**-2.20**	-1.73	**-2.01**	1.11
14	ALDH3B1	1.44	1.23	1.60	1.18	**-2.47**
15	ALDH4A1	1.24	-1.12	1.07	-1.47	1.99
16	ALDH7A1	-1.91	-1.54	-1.67	**-2.21**	1.69

*For genes exhibiting a fold change greater than 2, the p-value was less than 0.05.

Table S7. Transcript expression patterns of cadherins and cell adhesion molecules in hepatocyte organoids

Genes		Fold change values [$\log_2$ Each group/primary hepatocytes, (PH)]				
		Group 1	Group 2	Group 3	Group 4	Liver
1	ALCAM	-1.31	1.28	-1.09	-1.01	-1.52
2	BCAM	1.08	-1.16	1.56	**4.31**	**2.82**
3	CADM1	**-3.03**	**-2.60**	**-2.62**	-1.19	1.71
4	CADM2	**-227.74**	**-280.75**	**-277.16**	**-19.67**	-1.65
5	CADM3	**16.28**(p>0.05)	1.00	1.00	1.00	**97.71**
6	CADM4	**2.36**	1.85	**2.51**	**3.28**	1.63
7	CDH1	-1.45	-1.29	-1.08	1.34	-1.06
8	CDH11	1.00	**144.34**	**375.43**	**18112.87**	**901.16**
9	CDH13	-1.37	-1.16	**-2.09**	**4.62**	**2.77**
10	CDH19	1.00	1.00	1.00	1.00	**261.38**
11	CDH2	**-2.40**	-1.85	**-2.77**	**-3.05**	-2.02
12	CDH20	1.00	1.00	1.00	1.00	**82.83**
13	CDH23	**-2.06**(p>0.05)	**3.65**(p>0.05)	**2.80**(p>0.05)	**7.37**	**19.97**
14	CDH24	**115.57**	**131.31**	**322.88**	**742.27**	**834.21**
15	CDH3	**-17.80**(p>0.05)	-1.55	**5.86**	**25.98**	**6.15**
16	CDH4	1.00	1.00	1.00	1.00	**172.11**
17	CDH5	**-59.78**	**-73.53**	**-72.60**	**-72.75**	**194.93**
18	CDH6	1.00	**137.82**	**138.95**	**919.39**	**82.83**
19	CDH7	**-51.39**	-1.58	**-62.37**	-1.55	**25.75**
20	CDHR1	1.00	1.00	1.00	1.00	**75.39**
21	CDHR2	**21.13**	**12.77**	**62.84**	**14.24**	-8.33
22	CDHR3	1.00	**46.61**	1.00	1.00	**134.91**
23	CDHR4	1.94	-1.08	1.50	-1.17	**2.25**
24	CDHR5	1.07	-1.46	-1.71	**-3.71**	**7.87**
25	CEACAM1	**-3.23**	**-2.64**	**-3.33**	**-2.51**	1.13
26	CERCAM	**7.00**	**4.80**	**5.12**	**3.61**	-1.18
27	CHL1	1.00	1.00	**92.97**	**138.76**	**1503.75**
28	CXADR	**2.71**	**3.81**	**4.09**	**6.09**	-2.94
29	EPCAM	**2.02**	**2.77**	**9.60**	**5.84**	-5.47
30	ESAM	**-2.89**	**-166.78**	**-2.06**(p>0.05)	-1.00	**30.21**
31	ICAM1	**8.72**	**9.31**	**4.63**	**14.43**	**3.76**
32	ICAM2	-2.00	**-6.78**	**-4.90**	**-5.34**	**2.06**
33	ICAM3	**9.27**	**7.25**	**4.60**	**2.21**	**21.85**
34	L1CAM	1.00	**27.06**(p>0.05)	**244.05**	**73.16**	**3839.71**
35	MADCAM1	**12.07**	**6.64**	**-21.46**(p>0.05)	**9.20**	**48.01**
36	MCAM	-1.94	**7.30**	**-2.37**	**9.70**	**20.91**
37	NECTIN2	-1.16	-1.57	1.28	1.08	1.18
38	NECTIN3	-1.88	-1.33	-1.74	-1.61	-1.44
39	NECTIN4	**184.32**	**229.03**	**290.04**	**584.83**	**97.71**
40	NRCAM	**46.83**	**79.18**	**99.53**	**79.72**	**514.32**
41	PCDH1	**3.29**	1.99	**5.75**	**7.05**	**8.22**
42	PCDH12	1.00	1.00	1.00	**66.60**	**2046.82**
43	PCDH17	**-34.59**(p>0.05)	**-2.07**(p>0.05)	-1.54	**16.89**	**11.31**
44	PCDH18	**-311.72**	**-384.36**	**-379.44**	**3.61**	**25.60**
45	PCDH19	1.27	-1.49	1.67	1.28	**2.51**
46	PCDH7	1.00	1.00	**27.28**(p>0.05)	**519.23**	**201.86**
47	PCDHB6	1.16	-1.93	**-5.48**	1.68	1.15
48	PECAM1	-1.49	-1.36	-1.86	**-4.02**	1.64

49	SDK2	-1.15	-1.87	1.30	**2.38**	-1.62
50	VCAM1	**7.99**	**9.18**	**5.32**	**7.61**	**-2.08**

* Genes with fold changes > 2 were marked "p > 0.05" if p > 0.05.

Table S8. **Extracellular matrix transcript expression patterns in hepatocyte organoids**

Genes		Fold changes [log_2 Each group/primary hepatocytes, (PH, control)]				
		Group 1	Group 2	Group 3	Group 4	Liver
1	ADAMTSL1	**-42.99**(p>0.05)	**-52.81**	**-52.14**	**6.55**	**34.65**
2	ADAMTSL2	**-328.52**	**-405.08**	**-399.90**	**-5.48**	**53.00**
3	ADAMTSL3	**54.47**	**40.09**(p>0.05)	**53.55**	**374.91**	**566.39**
4	ADAMTSL4	**-4.46**	**-4.37**	**-2.99**	**-2.05**	**2.29**
5	ADAMTSL5	**9.50**	**12.64**	**42.91**	**32.08**	**7.78**
6	COL11A1	**7.35**	**6.94**	1.88	**69.00**	**30.13**
7	COL12A1	1.00	**40.09**(p>0.05)	**322.88**	**16978.01**	**3118.09**
8	COL13A1	1.00	**66.15**	1.00	**53.48**	**886.28**
9	COL14A1	**-15.44**	**-2.73**	-1.13	**23.48**	**15.61**
10	COL15A1	1.00	**40.09**	**198.07**	**15613.55**	**417.60**
11	COL16A1	**11.11**	**12.18**	**8.52**	**61.69**	**10.04**
12	COL18A1	**-4.50**	**-9.25**	**-8.80**	**-7.11**	**-2.89**
13	COL19A1	1.00	1.00	1.00	1.00	**365.53**
14	COL1A1	-1.75	**2.09**	**16.21**	**1424.05**	**43.97**
15	COL1A2	**-6.74**	-1.01	**4.52**	**404.34**	**35.26**
16	COL21A1	**5.39**	**7.16**	**93.28**	**26.80**	**32.12**
17	COL23A1	1.00	**14.03**(p>0.05)	1.00	**1385.14**	**120.03**
18	COL24A1	**-2.04**	-1.69	**-7.32**	**-5.22**	-1.83
19	COL26A1	**7.66**	**4.58**	**2.54**(p>0.05)	1.27	1.70
20	COL27A1	**12.46**	**11.18**	**7.09**	**14.89**	**2.40**
21	COL28A1	**8.30**	**9.23**	**9.41**	**6.40**	**-3.38**
22	COL3A1	**-2.68**	-1.40	**2.71**	**148.20**	**47.67**
23	COL4A1	-1.67	1.79	**3.74**	**97.71**	**21.91**
24	COL4A2	-1.22	**2.69**	**6.55**	**117.53**	**27.26**
25	COL4A3	1.00	1.00	**375.43**	**1627.85**	**774.69**
26	COL4A3BP	**-2.34**	-1.32	-1.48	-1.55	-1.77
27	COL4A4	1.72	1.94	**2.94**	**59.84**	**10.64**
28	COL4A5	**-5.97**	**-3.32**	**-6.16**	**5.69**	**9.07**
29	COL4A6	**-51.39**	**-63.17**	**-62.37**	-1.17	**30.11**
30	COL5A1	-1.32	1.41	**6.75**	**276.26**	**34.00**
31	COL5A2	-1.50	-1.15	1.33	**29.03**	**4.36**
32	COL5A3	**-68.18**	-1.41	**-4.00**	**14.63**	**30.95**
33	COL6A3	1.00	**352.83**	**690.74**	**96956.24**	**6012.00**
34	COL6A5	1.00	1.00	1.00	1.00	**6309.57**
35	COL6A6	**-185.75**	**-11.14**	**-4.22**	**-8.32**	**3.27**
36	COL7A1	1.14	1.02	**3.42**	**69.82**	**2.99**
37	COL8A1	**-4.80**	**-4.68**	**-7.76**	-1.71	**-10.84**
38	COL8A2	1.00	1.00	1.00	**27.24**(p>0.05)	**157.23**
39	COL9A3	**3.41**	**2.45**	1.63	**15.00**	-1.77
40	ELN	1.00	1.00	1.00	**315.87**	**1764.13**
41	FBLN1	-1.29	**-2.52**	-1.72	**-3.13**	**3.40**
42	FBLN2	**16.28**(p>0.05)	1.00	**171.79**	**1752.49**	**1883.16**
43	FBLN5	**-51.39**	**-63.17**	-1.04	**61.62**	**168.31**
44	FN1	-1.41	-1.63	-1.90	-1.29	-1.89
45	LAMA1	**53.02**	**66.64**	**5.35**	**30.17**	**2.33**(p>0.05)
46	LAMA2	**4.44**	**4.31**	**-2.37**	-1.48	**3.66**
47	LAMA3	**-2.50**	**-3.00**	1.94	**2.20**	**-5.71**
48	LAMA4	1.00	1.00	**14.14**(p>0.05)	**1745.93**	**1340.08**
49	LAMA5	**4.05**	**2.63**	**3.54**	**5.51**	-1.04

50	LAMB1	**4.09**	**4.98**	**3.83**	**5.42**	1.24
51	LAMB2	**-2.90**	**-3.27**	**-4.35**	**2.18**	**6.26**
52	LAMB3	**2.26**	1.26	**2.99**	**2.27**	**3.49**
53	LAMB4	**-3.28**	-1.88	**-4.38**	**-12.98**	-1.24
54	LAMC1	**4.06**	**4.62**	**4.71**	**8.02**	-1.09
55	LAMC2	**20.84**	**34.04**	**101.72**	**402.25**	**4.85**
56	LTBP1	1.27	**2.03**	**3.93**	**36.66**	**72.75**
57	LTBP2	-1.53	-1.58	**2.33**	**36.87**	**42.89**
58	LTBP3	1.05	1.19	1.37	**7.14**	**6.28**
59	LTBP4	**-2.09**(p>0.05)	**-10.64**	-1.92	1.46	**43.54**
60	MMP2	1.13	**5.60**	**26.82**	**984.45**	**39.85**
61	SP1	1.10	1.29	1.32	1.67	1.36
62	SP3	-1.88	-1.09	-1.53	-1.36	**-2.01**
63	TGFB1	**31.93**	**32.02**	**38.06**	**136.62**	**70.76**
64	TGFBR1	**4.58**	**7.39**	**8.21**	**13.59**	1.76
65	TGFBR2	1.31	1.16	**2.07**	**2.92**	**3.13**
66	TIMP1	**4897.06**	**6418.56**	**4270.85**	**14695.16**	**730.06**
67	TIMP2	1.01	**4.02**	**11.02**	**460.56**	**71.13**
68	TIMP3	1.39	1.58	1.56	**8.48**	1.43

*Genes with fold changes greater than 2 were labeled with "p > 0.05" next to the gene name if their p-value exceeded 0.05

Supplementary Figure Legends

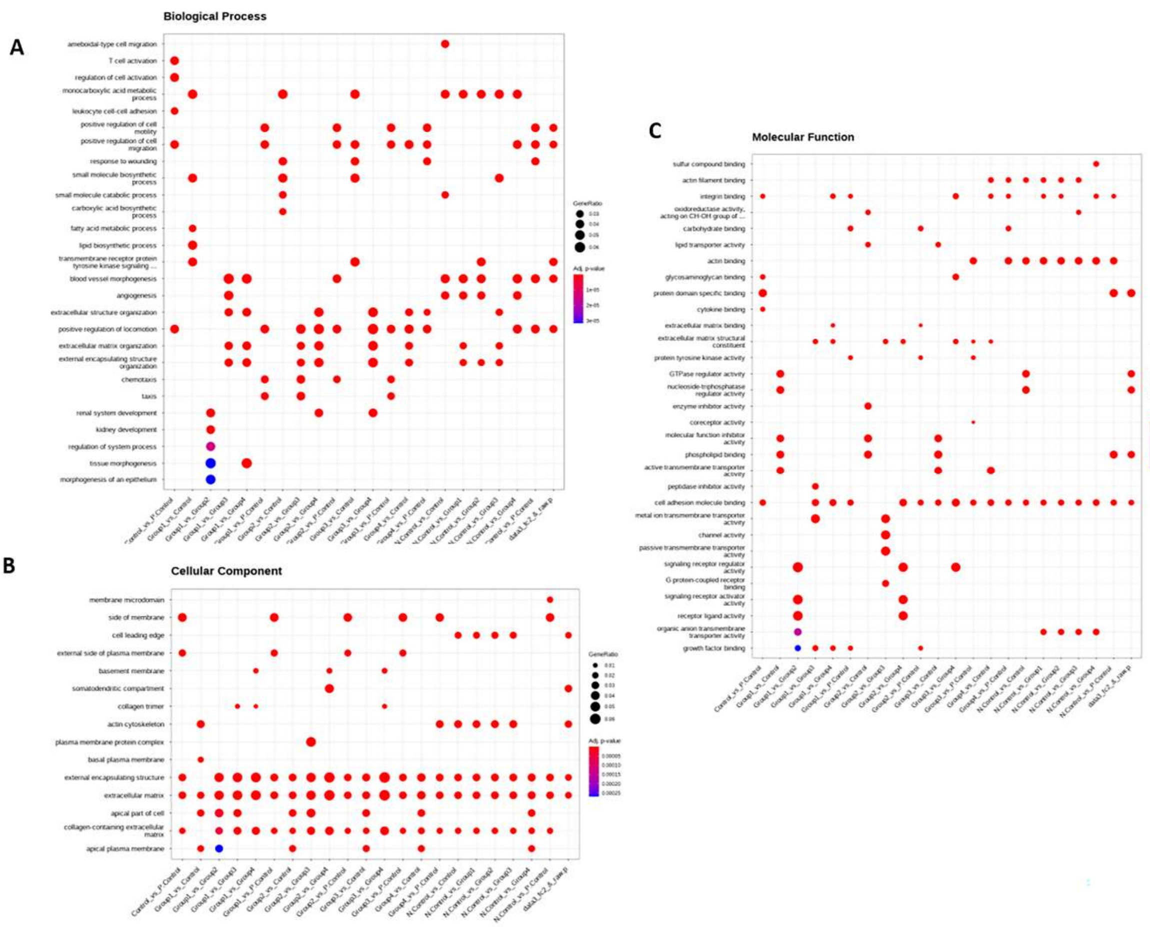

Figure S1. Gene Ontology (GO) analysis results of hepatocyte organoids (HOs). Analysis of (A) biological processes, (B) molecular functions, and (C) cellular components. The groups were divided as follows: at passage 0, 14-day-old hepatocyte organoids (HOs) cultured alone (Group 1, G1) or co-cultured with A-MSCs (Group 2, G2), and at passage 2, 42-day-old HOs cultured alone (Group 3, G3) or co-cultured with A-MSCs (Group 4, G4). Liver, primary hepatocytes (PH), and ear fibroblasts (EF) were used as the positive controls (PC), controls (C), and negative controls (NC), respectively.

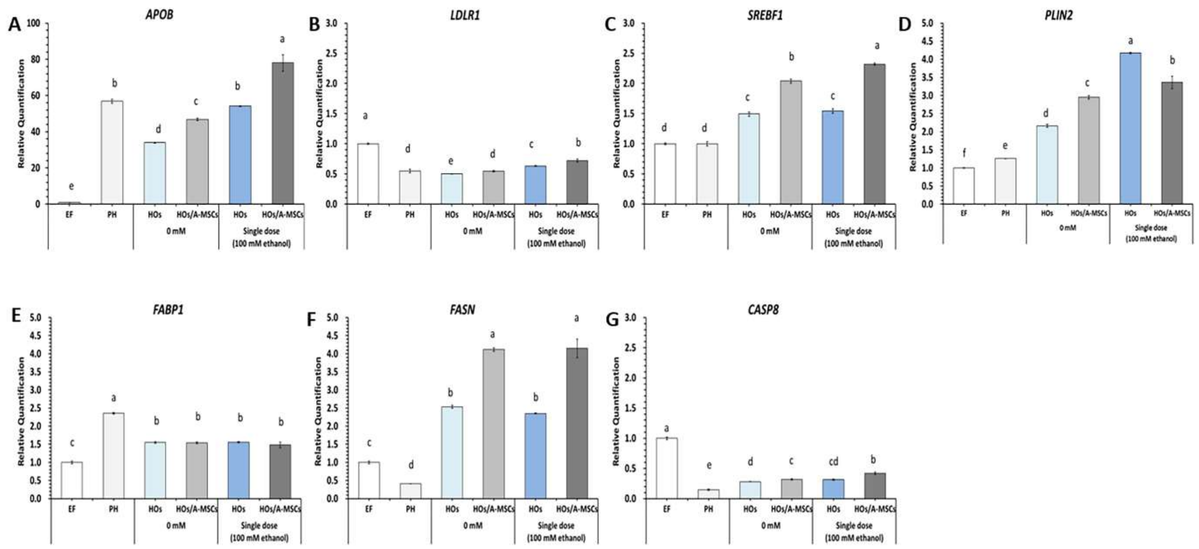

Figure S2. Pathway analysis of lipid and cell death responses to single-dose ethanol exposure in hepatocyte organoids (HOs). On day 11, HOs co-cultured with A-MSCs (HO/A-MSCs) and HOs cultured alone were incubated in fresh organoid culture medium with or without 100 mM ethanol for an additional 72 h. mRNA was extracted from HOs for real-time PCR analysis of *APOB* (A), *LDLR1* (B), *SREBF1* (C), *PLIN2* (D), FABP1 (E), *FASN* (F), and *CASP8* (G) gene expression. The data are presented as the mean relative quantification (RQ) ± maximum and minimum values, normalized to that in ear fibroblasts (EF). Statistical significance was assessed using a one-way analysis of variance (ANOVA) with $^{a\text{-}e}$ $p < 0.05$ as the threshold after five repetitions. PH, primary hepatocytes isolated from pig livers.

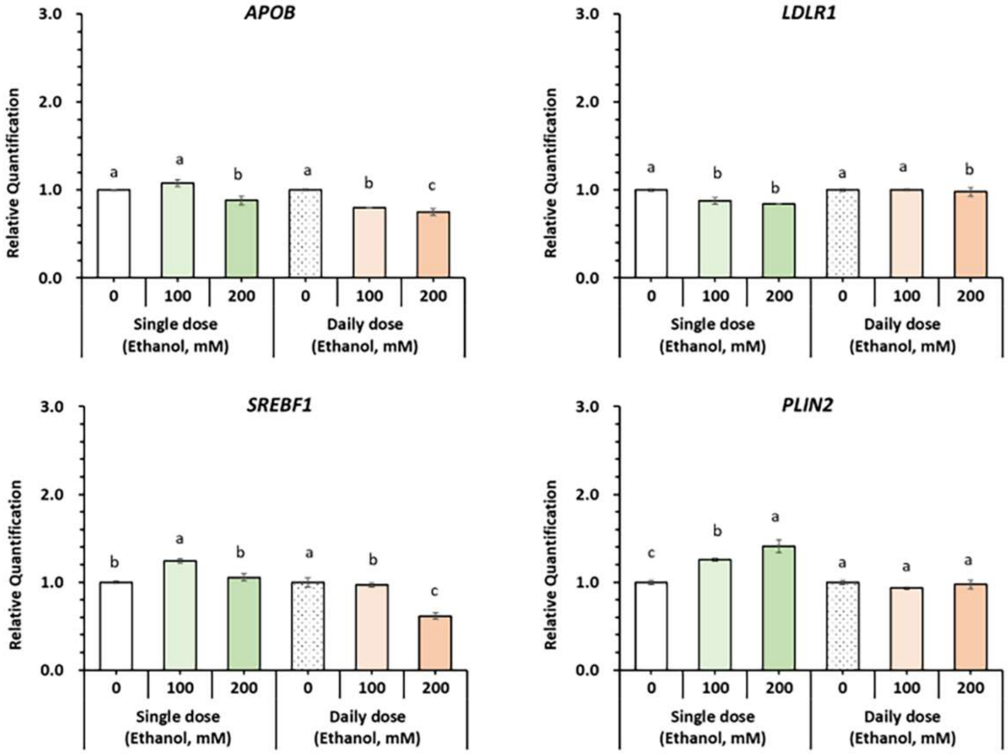

Figure S3. Effect of ethanol exposure on the expression of lipid metabolism and apoptosis genes in A-MSCs. A-MSCs were seeded at a density of 4,000 cells/well in a 24-well plate and exposed to a single or daily dose of 0, 100, or 200 mM ethanol for three days. The expression of genes related to lipid metabolism, *APOB*, *LDLR1*, *SREBF*1, and *PLIN2*, were analyzed. The data are presented as the mean relative quantification (RQ) ± the maximum and minimum values, normalized to that in A-MSCs without ethanol for RQ in each of the single and daily treatment groups. Statistical significance was assessed using one-way analysis of variance (ANOVA) with $^{a\text{-}c}$ $p < 0.05$ as the threshold after five repetitions.
